# Release of Periplasmic Nucleotidase Induced by Human Antimicrobial Peptide in *E*. *coli* Causes Accumulation of the Immunomodulator Adenosine

**DOI:** 10.1371/journal.pone.0138033

**Published:** 2015-09-15

**Authors:** Andreia Bergamo Estrela, Patrick Türck, Elaine Stutz, Wolf-Rainer Abraham

**Affiliations:** Chemical Microbiology, Helmholtz Centre for Infection Research, Inhoffenstrasse 7, 38124, Braunschweig, Germany; University of Colorado Denver, UNITED STATES

## Abstract

Previous work by our group described that human β-defensin-2 induces accumulation of extracellular adenosine (Ado) in *E*. *coli* cultures through a non-lytic mechanism causing severe plasmolysis. Here, we investigate the presence of AMP as a direct precursor and the involvement of a bacterial enzyme in the generation of extracellular Ado by treated bacteria. Following hBD-2 treatment, metabolites were quantified in the supernatants using targeted HPLC-MS/MS analysis. Microbial growth was monitored by optical density and cell viability was determined by colony forming units counts. Phosphatase activity was measured using chromogenic substrate pNPP. The results demonstrate that defensin-treated *E*. *coli* strain W releases AMP in the extracellular space, where it is converted to Ado by a bacterial soluble factor. An increase in phosphatase activity in the supernatant was observed after peptide treatment, similar to the effect of sucrose-induced osmotic stress, suggesting that the periplasmic 5'nucleotidase (5'-NT) is released following the plasmolysis event triggered by the peptide. Ado accumulation was enhanced in the presence of Co^2+^ ion and inhibited by EDTA, further supporting the involvement of a metallo-phosphatase such as 5’-NT in extracellular AMP conversion into Ado. The comparative analysis of hBD-induced Ado accumulation in different *E*. *coli* strains and in *Pseudomonas aeruginosa* revealed that the response is not correlated to the peptide's effect on cell viability, but indicates it might be dependent on the subcellular distribution of the nucleotidase. Taken together, these data shed light on a yet undescribed mechanism of host-microbial interaction: a human antimicrobial peptide inducing selective release of a bacterial enzyme (*E*. *coli* 5'-NT), leading to the formation of a potent immunomodulator metabolite (Ado).

## Introduction

Multicellular organisms, including humans, are inhabited by a huge amount of microbes, collectively called microbiota. The relationship between animals and the microbial community living within them has an ancient evolutionary origin; the importance of this interaction is evident in the multiple roles attributed to the microbiota in their hosts' health status [1]. A complex balance involving commensal, mutualistic and infectious miccroorganisms must be kept to ensure harmonized co-existance: on the one hand several bacteria perform important functions, *e*.*g*. delivering nutrients including vitamins to the host; on the other hand, some can act as opportunistic pathogens. Host-microbial communication is a central piece in maintaining the homeostasis of abundant and diverse microbial communities such as the human intestinal microflora [2]. Disturbances in this delicate trade are known to have several clinical implications for the host as has been demonstrated for obesity [3], allergy [4] or inflammatory bowel disease (IBD) [5] and many more correlations are emerging [6]. Thus, a multitude of mechanisms are employed, both by the host and the microbiota, to control detrimental microbes and simultaneously foster beneficial ones [7].

One important strategy is the production of antimicrobial peptides. Human antimicrobial peptides belong mainly to the cathelicidin and defensin families [8]. Defensins have around 30–50 amino acids, are stabilized by disulfide bridges and grouped into α- or β-defensins. Their production is differentially controlled, leading to specific mixtures of defensins in different organs. In the intestine, Paneth cells mainly release α-defensins into the crypts, while colonic epithelial cells excrete β-defensins [9] [10]. Being part of the first-line of defense in the intestinal mucosa, the epithelial antimicrobial peptides act in shaping the microbial community and controlling undesired infection by potential pathogens. In addition to their role as antimicrobials, some peptides are emerging as important immune regulators, orchestrating cellular responses and modulating inflammatory processes [11]. The action of defense peptides can also trigger responses from the affected bacteria, and their immunomodulatory traits, combined to moderate antimicrobial activity, offers efficiency against infections with lower risk of resistance development [12] [13].

Previous work by our group has demonstrated that a non-lytic mechanism of action of human β-defensin-2 (hBD-2) can trigger membrane dissociation (plasmolysis) and extracellular accumulation of adenosine (Ado) from the bacterium *Escherichia coli* [14]. Given the multitude of biological activities attributed to Ado signaling during inflammation, this novel perspective placed hBD-2 as a component of the host-microbial communication arsenal, able to elicit from the bacterial target the release of an important immunomodulatory molecule. In addition, this finding has a potential for the treatment of inflammatory bowel diseases (IBD) [15] where, for instance, methotrexate, which induces Ado release, is commonly applied as an anti-inflammatory agent for remission and maintenance therapy in IBD patients [16]. Production of Ado on site by gut bacteria when challenged with defensin could overcome problems such as incomplete tissue selectivity of drugs and the short half-life of adenosine in the human body [17].

In the present study, our aim was to investigate the possible involvement of a bacterial enzymatic activity in the generation of adenosine following hBD-2 treatment, and its correlation to the plasmolysis induced in treated bacteria.

## Material and Methods

### Bacteria, culture conditions and defensin treatment

The strains used in this work are presented in Table 1. *E*. *coli* Nissle 1917 and LF82 [18] were kindly provided by Dr. Gabriella Molinari (Helmholz-Centre for Infection Research). *E*. *coli* 7145A and HZI 2–6 were isolated by Dr. Alexander Swidsinski (Medical Clinic of the Humboldt-University Berlin Charité) and Dr. Dieco Würdemann (Helmholz-Centre for Infection Research), respectively, from biopsies from patients with intestinal inflammation at the University Hospital Schleswig-Holstein, Kiel. The DSM strains were purchased from the German Collection of Microorganisms and Cell Cultures.

**Table 1:** Bacterial strains used in this study.

Strain	Description
*Escherichia coli* strain W	DSM1116; quality control strain for antibiotics
*E*. *coli* Nissle 1917	probiotic
*E*. *coli* 7145A	clinical isolate
*E*. *coli* HZI 2–6	clinical isolate
*E*. *coli* LF82	adherent-invasive strain; pathogenic [18]
*Pseudomonas aeruginosa*	DSM19882 (PA14)
*Bacillus cereus*	DSM31

Bacteria were cultured in minimal mineral medium (MgSO_4_ 0.02 g∙L^-1^, citric acid 0.2 g.L^-1^, K_2_HPO_4_ 1 g∙L^-1^, NaNH_4_HPO_4_ 0.32 g∙L^-1^), supplemented with glucose 0.2%. For the typical assay, cultures (160 μL) were inoculated at optical density (OD or A_600 nm_) 0.005 in 100-well honeycomb microplates (Oy Growth Curves Ab Ltd, Finland) and kept at 37°C under agitation. Growth was monitored by OD measurement every 15 min in an automated system (Bioscreen C, Oy Growth Curves Ab Ltd). Alternatively, for extracellular phosphatase activity determination, bacteria were cultured (20 mL) in Erlenmeyer flasks and OD monitored every hour in a cuvette spectrophotometer (Eppendorf, Hamburg, Germany).

The addition of antimicrobial peptide was performed when cell density reached OD ~ 0.1. A stock solution of human β-defensin-2 (hBD-2) (Peptide Institute Inc., Osaka, Japan) (100 μg∙mL^-1^) was added at a 1:4 (v/v) ratio (final concentration 20 μg∙mL^-1^). Untreated controls were handled in all experiments by the addition of an equal volume of sterile water. Samples were collected at specified time-points and cells were separated from supernatants using microtube-filters (0.2 μm pore).

### Osmotic shock induction

When indicated, alternatively to the antimicrobial treatment (following identical culture conditions), cultures were submitted to sucrose-induced osmotic shock. For MS analysis, osmotic shock-induced plasmolysis was achieved following the protocol adapted from Scheie (1969), where cell were harvested by centrifugation (10 min 4,000 rpm) and the pellets were resuspended in 0.8 M sucrose. Samples were collected by filtration as described above, 5 min or 2 h after the treatment. For evaluation of the enzymatic activity, the osmotic shock protocol employed was as described by Broad and Smith, 1981 [19]. Briefly, cells were harvested by centrifugation (20 min, 4,000 rpm), the pellet resuspended in 20% sucrose, 1 mM EDTA in Tris buffer 0.03 M, pH 7,3 and incubated for 5 min at room temperature under agitation. Cell suspension was again centrifuged, the pellet resuspended in cold water and incubated for further 5 min at room temperature under agitation. Finally, this suspension was centrifuged and the supernatant (referred to as osmotic fluid) was collected for analysis.

### Liquid chromatography and mass spectrometry

Detection of adenosine nucleoside and adenosine monophosphate nucleotide was performed by targeted tandem mass spectrometry (multiple reaction monitoring mode) in a 6460 TripleQuad LC-MS system (Agilent Technologies, Santa Clara, CA, USA) as described [14].

### Colony forming units (CFU) counts

To evaluate bacterial susceptibility to the antimicrobial treatment, cultures were serially diluted (10-fold steps) in sterile physiological solution (0.9% NaCl) and plated (100 μL) in triplicates onto LB agar plates. After 24 h at 37°C, plates containing between 30 and 300 visible colonies were used for CFU counting. Lethality was calculated as the difference in the number of viable cells 2 h after defensin treatment, relative to the total viable cells before the treatment.

### Phosphatase activity determination

Enzyme activity was investigated using the chromogenic substrate p-nitrophenylphosphate (pNPP). Previous to the analysis, samples were dialyzed in the reaction buffer (sodium acetate 100 mM, CoCl_2_ 5 mM, CaCl_2_ 20 mM, pH 6), and concentrated 20-fold in a Centricon device (MWCO 3,000). The reaction mixture containing 30 μL of buffer, 50 μL of sample and 20 μL of substrate (pNPP 200 mM, final concentration 40 mM in 100 μL) was pipeted in microtitre plates and incubated at 37°C under agitation in Bioscreen C system. The formation of the yellow-coloured dephosphorylated product was monitored by measuring the absorbance at 405 nm every 30 min for up to 24 h. Initial reaction rate (V_0_) was calculated as the slope of the tangent line at time = 0 on the absorbance *versus* time curve.

### Statistical analyses

Data obtained in at least three biological replicates were tested for statistical differences between the means using the software SigmaPlot 13.0 (Systat Software, Inc., Chicago, IL, USA). The following tests were employed: t-test for pairwise single comparison (treated *vs*. untreated); one-way analysis of variance (ANOVA) using Holm-Sidak test for pairwise multiple comparisons when normality held; or, for non-normally distributed data, Kruskal-Wallis one-way ANOVA on Ranks using either Tukey test or Dunn's test for pairwise multiple comparisons on equal or unequal sample sizes, respectively.

## Results

### Supernatants from defensin-treated bacteria generate Ado from AMP

**Fig 1:**
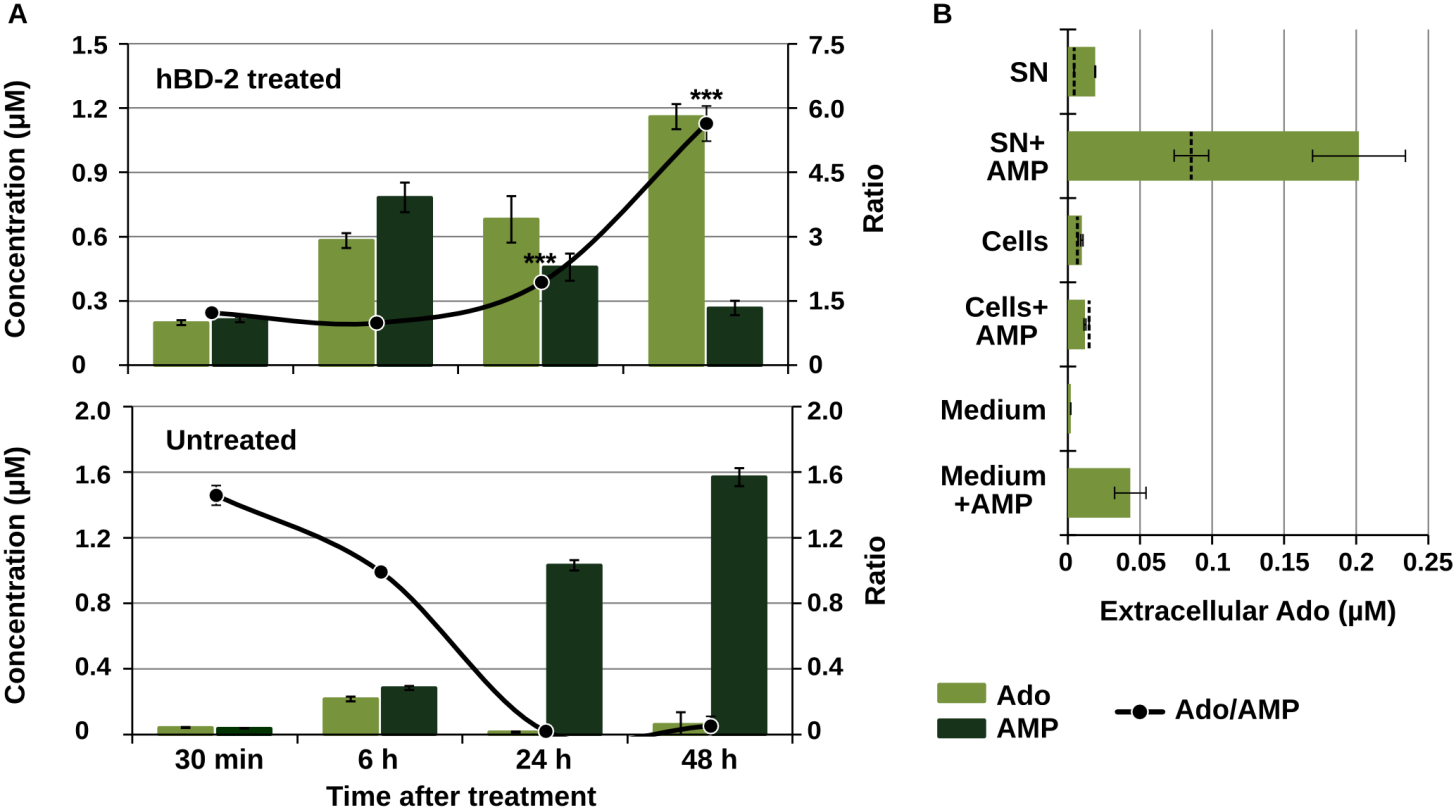
Extracellular AMP degradation as the source for extracellular Ado in defensin-treated bacteria. **A:** extracellular concentration of Ado and AMP in *E*. *coli* (DSM 1116) cultures over time after hBD-2 treatment (20 μg.mL^-1^, upper panel) or in untreated cultures (lower panel). Supernatants were sampled by filtration at the indicated time-points and analyzed by targeted LC-MS/MS. Data are mean±SD (n = 3). *** p<0.001 in t-test (treated *vs*. untreated). **B:** effect of exogenous AMP on Ado accumulation by soluble (SN) versus cell-bound (Cells) fractions of defensin-treated *E*. *coli* (DSM 1116) cultures. The cultures were handled as described in Material and Methods up to O.D. 0.1. The two fractions were separated by filtration immediately after hBD-2 (20 μg.mL^-1^) addition and further incubated for 2 h in the presence or absence of exogenous AMP (1μg.mL^-1^), before analysis by targeted LC-MS/MS. Dashed lines represent levels of extracellular Ado in untreated controls. Sterile medium with hBD-2 (20 μg.mL^-1^) was used as a control for spontaneous AMP conversion during the experiment. Data are mean±SD (n = 2).

In our previous work, we had described the extracellular accumulation of Ado induced by defensin treatment in *E*. *coli*, as well as the presence of AMP as one of the purine-related metabolites released by treated bacteria. To gain some insight on the role of AMP as a precursor for the accumulated Ado, the levels of extracellular AMP accumulated by *E*. *coli* (DSM 1116) submitted to defensin treatment were measured by targeted LC-MS/MS at different time-points after the antimicrobial treatment. The results demonstrated an accumulation of both metabolites during the first six hours in hBD-2 treated cultures (Fig 1A upper panel). In the subsequent 24 h and 48 h, it was observed that the accumulated AMP was gradually degraded, concomitantly with an increase in extracellular Ado concentration, as opposed to the dynamics shown by untreated bacteria (Fig 1A, lower panel).

To further support the notion that AMP acts as a direct precursor in the extracellular generation of Ado, it was tested whether the addition of exogenous AMP would influence Ado formation by defensin-treated bacteria. Cells were separated from the supernatants by filtration (0.2 μm pore) immediately after treatment; the soluble filtrate and the cell-bound components (resuspended in 200 μL sterile medium) were assayed separately in the presence or absence of exogenous AMP for 2h. This experiment was designed to verify the presence of an AMP-converting activity and to investigate whether it was attributable to a secreted or a cellular factor. Fig 1B shows that Ado accumulated in cell-free supernatant (“SN”) of hBD-treated cultures in comparison to untreated control; the addition of exogenous AMP dramatically increased the concentration of Ado in these samples: AMP was converted into Ado in the soluble fraction of untreated cultures, however, this activity was higher when bacteria were exposed to hBD-2. Ado accumulation was not observed in the cell-bound component (“Cells”), regardless of the presence of added AMP. Ado was also generated from exogenous AMP in sterile medium, however spontaneous conversion was not high enough to account for the results observed in the supernatants. The data indicate that a bacterial factor released upon hBD-treatment is responsible for converting AMP into Ado in the extracellular space.

### A periplasmic phosphatase activity is the soluble bacterial factor responsible for Ado formation

**Fig 2:**
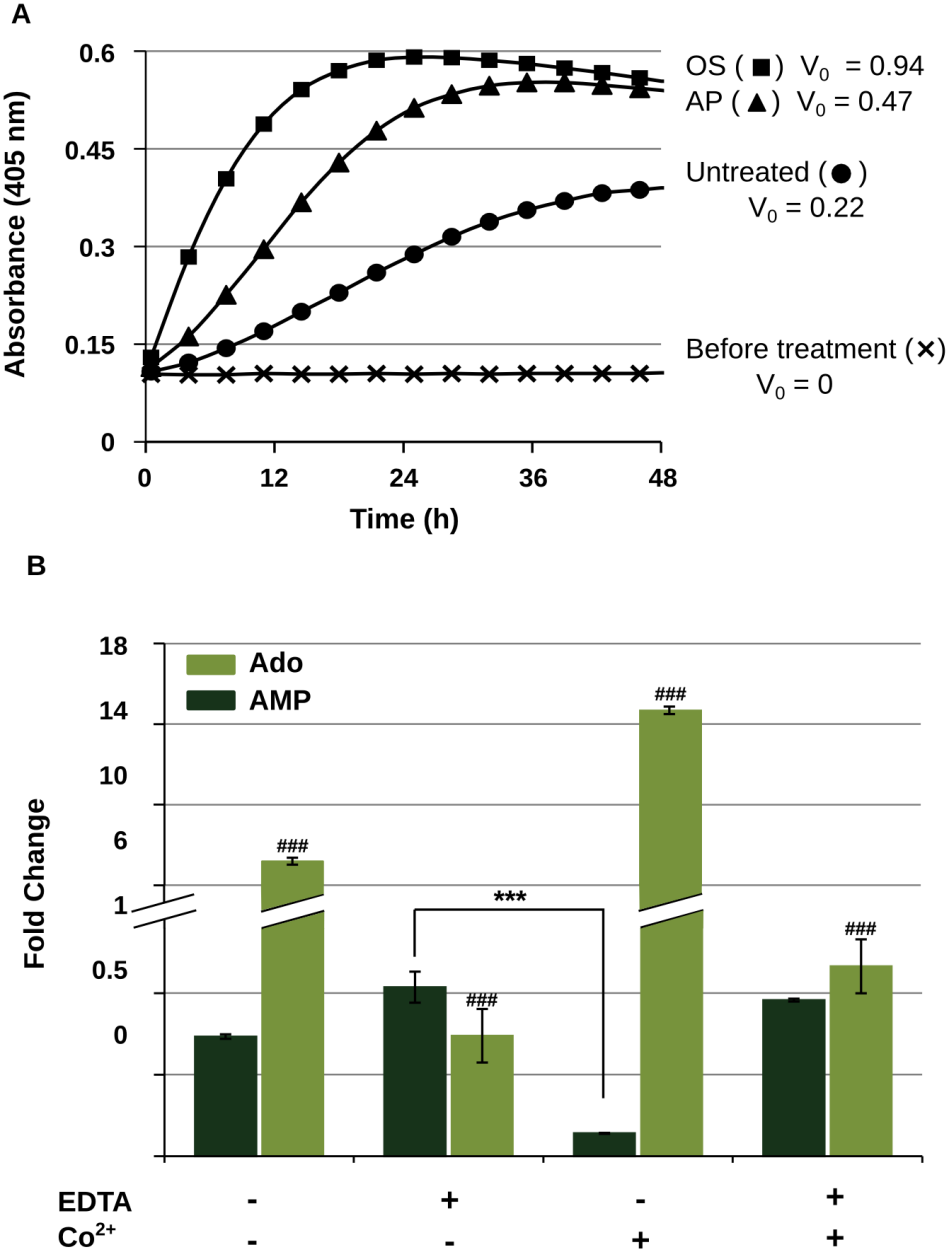
Involvement of metallo-phosphatase activity in the response of *E*. *coli* to defensin treatment. **A:** Extracellular phosphatase activity in *E*. *coli* (DSM 1116) cultures. Supernatants were sampled before peptide addition (crosses) and after 4 h in the absence (untreated, circles) or presence of antimicrobial peptide (SMAP; 25 μg∙mL^-1^, AP, triangles), or after sucrose-induced osmotic shock treatment (OS, squares). Samples were tested for the presence of phosphatase activity using pNPP (40 mM) as chromogenic substrate and the initial reaction rate (V_0_) was defined as the slope of the kinetic curve in the linear portion. **B:** Effect of metal chelating agent (EDTA) and Co^2+^ ion on extracellular AMP and Ado levels in *E*. *coli* (DSM 1116) cultures after hBD-2 treatment. Supernatants were collected by filtration 6 h after the treatment and incubated for further 18 h in the presence or absence of EDTA (2 mM) and/or CoCl_2_ (5 mM). Samples were analyzed by targeted LC-MS/MS. Data are presented as fold-change in metabolite concentration from the time supernatants were collected to the end of the incubation period (mean±SD; n = 6 for first and forth x-axis categories and n = 5 for second and third x-axis categories). ### p<0.001 in all pairwise comparisons and *** p<0.001 in the indicated pairwise comparison, as analyzed by ANOVA followed by multiple comparison procedure (see Material and Methods for detail).

The presence of AMP-dephosphorylating enzymes was investigated in the supernatants from *E*. *coli* (DSM 1116) cultures submitted or not to antimicrobial peptide (AP) treatment, using a chromogenic substrate to detect phosphatase activity (pNPP). Fig 2A shows the formation of the yellow-colored pNPP reaction product over time in the supernatants samples from each experimental condition, and presents the corresponding initial reaction rate (V_0_) for comparison. As a positive control, the supernatants resulting from an osmotic shock treatment (OS) were tested, which was described in the literature as a method to extract periplasmic nucleotidase from *E*. *coli* [19]. For technical reasons, the culture volume required to apply the osmotic shock protocol was higher than the typical defensin-treatment microplate assay (see Material and Methods). We then sought to adjust the volume of peptide-treated samples accordingly, at the same time avoiding the substantial increase in costs that would result from using large quantities of synthetic human β-defensin-2. Therefore, we have decided to use a substitute, less expensive product, sheep-myeloid antimicrobial peptide 29 (SMAP-29); this is a mammalian α-helical cathelicidin which was shown to elicit an extracellular Ado accumulation response in *E*. *coli* similar to hBD-2 [14]. SMAP-29 was used for the experiments presented in Fig 2A.

Phosphatase activity was absent in culture supernatants before the treatment (Fig 2A, crosses). A basal level of extracellular activity was observed in untreated cultures 4 h later (Fig 2A, circles). In turn, phosphatase activity found in the AP-treated culture supernatants 4 h after peptide addition was higher (Fig 2A, triangles), approaching the levels found in cultures submitted to osmotic shock (Fig 2A, squares). A validation test using the microplate setup confirmed that SMAP-29 and hBD-2 have similar effect upon *E*. *coli* regarding extracellular phosphatase activity (V_0_ 0.23 and 0.31 for SMAP-treated and hBD-2-treated bacteria, respectively). This pointed to an osmotic-shock-like (plasmolysis) effect of peptide treatment on enzyme release and/or activity, suggesting the involvement of a periplasmic phosphatase in extracellular AMP conversion by hBD-2 treated bacteria.

*E*. *coli* periplasmic 5' nucleotidase is a metallohydrolase, and additional evidence of its role on Ado generation was sought by testing the effect of a metal chelating agent (EDTA) and the cofactor Co^2+^ ion on AMP degradation in defensin-treated bacterial supernatants. The supernatants were sampled by filtration 6 h after hBD-2 addition, when extracellular levels of endogenous AMP were the highest (see Fig 1A). Metabolites levels were measured immediately upon sampling (pre-incubation) and after 18 h of incubation in the presence or absence of the inhibitor and/or activator cofactor. Fig 2B shows the fold change on AMP and Ado concentration from the onset to the end of incubation time,. In the control condition (the absence of either inhibitor or activator), we observed a decrease in AMP concentration paralleled by a 7-fold increase in Ado levels. When EDTA was present, AMP levels were unchanged and Ado formation was not observed. In contrast, with addition of the metal cofactor, AMP consumption and the generation of Ado were dramatically enhanced, an effect counteracted by simultaneous addition of the inhibitor.

**Fig 3:**
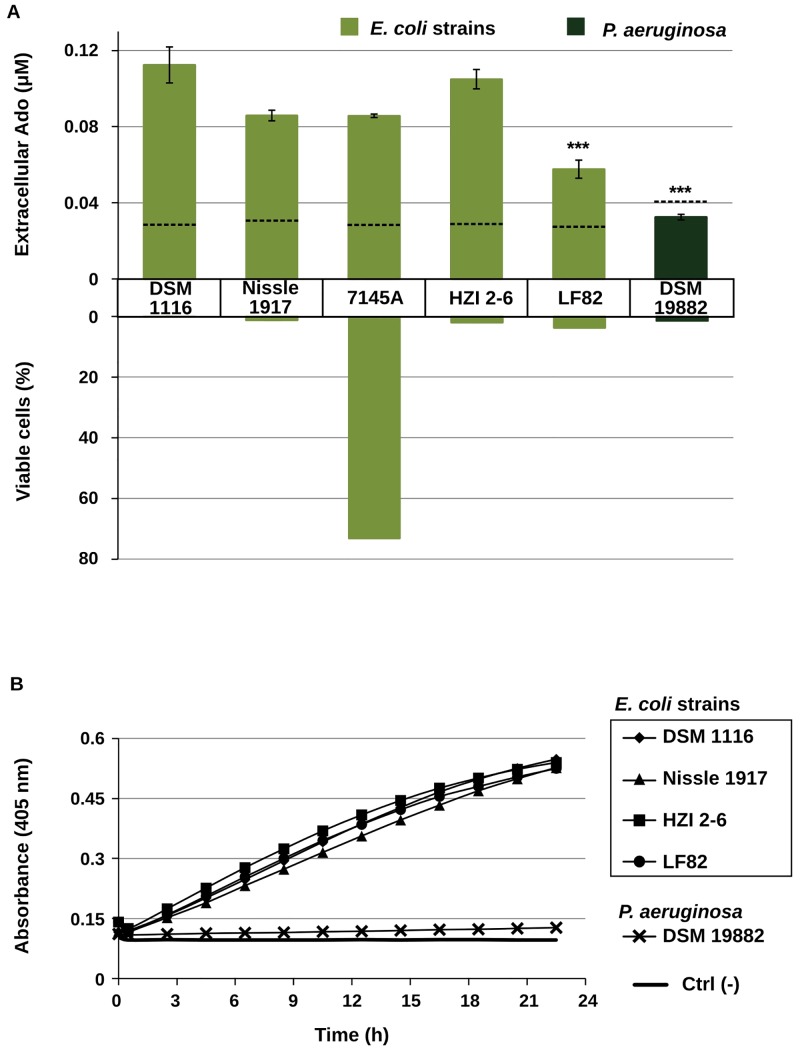
Strain- and species-specific differences in bacterial response to defensin and levels of periplasmic phosphatase activity. **A:** Extracellular adenosine concentration produced by different bacteria and cell viability in each culture after hBD-2 (20 μg∙mL^-1^) treatment. Cultures start OD was 0.02. Supernatants were sampled 2 h after peptide addition and analyzed by targeted HPLC-MS/MS for the presence of Ado (bar chart, mean ± SD, n = 6; ***<0.001 compared to DSM1116 by ANOVA followed by multiple comparison procedure. Refer to Material and Methods for detail). Dashed lines represent levels of extracellular Ado in untreated controls. Susceptibility was evaluated by CFU counts in LB agar, and calculated as the percentage of viable cells remaining 2 h after the treatment, in respect to the number of viable cells in each culture immediately before peptide addition. **B:** Periplasmic phosphatase activity in different bacteria submitted to osmotic shock. Supernatants were sampled after sucrose-induced osmotic shock treatment and tested for the presence of phosphatase activity using pNPP (40 mM) as chromogenic substrate. Ctrl. (-) corresponds to the reaction in the absence of supernatants.

To further characterize the involvement of a periplasm-located nucleotidase activity in the extracellular response triggered by defensin, we have compared six different bacterial strains. First they were analyzed for their response to hBD-2 treatment, regarding survival and extracellular Ado concentration produced 2 h after peptide addition (Fig 3A). No correlation between the lethality of the treatment and the levels of Ado released could be established. The accumulation of Ado in the medium was variable among the different strains, but independent of the percentage of viable cells in the cultures. For example, the *E*. *coli* isolate 7145A, despite being more resistant to the treatment, produced similar levels of extracellular Ado as the susceptible *E*. *coli* Nissle 1917 strain. Interestingly, the response of a different Gram-negative species, *Pseudomonas aeruginosa*, revealed it was unable to produce extracellular Ado in response to defensin, even though the treatment was equally effective in reducing cell viability.

We then analyzed the same strains for the levels of periplasmic phosphatase activity extracted using osmotic-shock method (Fig 3B). It was possible to detect similar levels of the enzyme in all *E*. *coli* strains (Fig 3B, closed symbols); in contrast, no phosphatase activity was found in *P*. *aeruginosa* supernatants following osmotic-shock, as demonstrated by its inability to metabolize the chromogenic substrate (Fig 3B, crosses), comparable to the negative control. The data pointed to a correlation between the presence and/or activity of periplasmic nucleotidase and the accumulation of extracellultar Ado after defensin treatment. In addition, we have observed that *Bacillus cereus*, a gram-positive bacterium with reduced or absent periplasmic space, presented 10-fold lower Ado accumulation response to defensin treatment when compared to *E*. *coli* (0.06 μM *versus* 0.71 μM, 2 h after hBD-2 20 μg/mL addition), providing further support to our results.

## Discussion

The accumulation of extracellular adenosine, a potent immunomodulator, by bacteria when confronted with antimicrobial peptide activity is a phenomenon only recently described. The data presented here extend our emerging knowledge about the mechanisms leading to this response. The presence of extracellular AMP in *E*. *coli* cultures after defensin treatment had been documented in our previous study, and it was here further characterized as a precursor in the generation of adenosine by cultures treated with defensin. AMP was rapidly produced by treated cultures in the first 6 h and its subsequent depletion was found to correlate with formation of Ado (Fig 1A). In contrast, intact *E*. *coli* accumulated extracellular AMP, a reaction that might be related to RNA degradation and purine excretion in stationary-growth phase of *E*. *coli* [20, 21]. In addition, the presence of exogenous AMP enhanced Ado accumulation after hBD-treatment exclusively in the soluble fraction of the cultures (Fig 1B). Taken together, the results confirm AMP as a direct precursor for Ado generation and point to the involvement of a bacterial factor secreted upon peptide addition.

In intact Gram-negative cells, the flux of purine nucleotides across the outer membrane is mediated by porines [22]. In the periplasmic space, nucleotides are then metabolized to adenine or taken up as Ado via concentrative nucleoside transporters in the cytoplasmic membrane. In *E*. *coli*, three periplasmic enzymes have been found involved in purine metabolism: adenylate kinase (E.C. 2.7.4.3), purine-nucleoside phosphorylase (E.C. 2.4.2.1) and 5’ nucleotidase (E.C. 3.1.3.5) [22]. The latter is a metalloenzyme performing dephosphorylation of AMP to Ado. It is readily and selectively released from the periplasmic space to the extracellular medium in situations of osmotic stress and plasmolysis [19,23]. Our previous findings demonstrated that hBD-2-treated *E*.*coli* undergoes plasmolysis, releasing nucleotide-related metabolites while retaining intracellular macromolecules [14]. This scenario could account for the presence of periplasmic 5’ nucleotidase in the extracellular medium, which in turn could be the soluble bacterial factor responsible for converting AMP into Ado after bacteria had been challenged with defensin. We verified this hypothesis as we found phosphatase activity in the supernatants of peptide-treated bacteria at higher levels than in untreated controls, closer to the activity obtained by a sucrose-induced osmotic stress used to extract periplasmic nucleotidase from *E*.*coli* (Fig 2A). Furthermore, the negative impact on Ado generation of metal chelator EDTA, as well as the activation effect of Co^2+^ ion (Fig 2B), supported the involvement of a metalloenzyme such as 5’nucleotidase in the bacterial response to defensin.

In this context, it would be reasonable to expect that different bacteria would respond differently to defensin treatment, according to their characteristics of periplasmic enzyme content. We have found the accumulation of Ado in response to defensin in five different *E*. *coli* strains, including probiotic, pathogenic and clinical isolates, but not in *P*. *aeruginosa* (Fig 3) or in gram-positive *B*. *cereus*. Although there is variability in the amount of extracellular Ado among the responding strains, this difference did not correlate with the strain’s susceptibility to the treatment. On the other hand, the extracellular Ado response is only present in gram-negative strains releasing phosphatase activity under osmotic stress. Altogether, these data offer two sets of information discussed below.

First, the presence and prompt release of periplasmic 5’ nucleotidase is important for the bacteria to respond to defensin with Ado accumulation. It was reported that in *P*. *aeruginosa*, less than 50% of the cellular nucleotidase pool is found in the periplasm, and that the enzyme is bound to this subcellular compartment in a way different from other enzymes [24–27]. In contrast, in *E*. *coli*, more than 90% of the nucleotidase activity corresponds to the periplasmic fraction, and indications have been found that the elongated form of *E*. *coli* 5’nucleotidase may facilitate its release from cells in conditions where other globular proteins of similar molecular mass would be retained [19]; this is consistent with the observation that this enzyme is present in the supernatants of defensin-treated cultures even when there is no increase in the total extracellular protein content. In addition, the absence of Ado accumulation a the gram-positive species, devoid of periplasmic space, corroborates the idea that periplasmic location of the enzyme is determinant for its release after defensin stress.

Second, the accumulation of Ado is not solely a collateral effect of cell death. Since the release of periplasmic nucleotidase can occur, as mentioned, independently of a lysis event, and the response is observable even when a high proportion of the cells remain viable after the defensin treatment (the case in *E*. *coli* isolate 7145A), it indicates that effective cell killing by the peptide is not required for the raise in extracellular Ado concentration. Instead, it corroborates the hypothesis [14] that optimal peptide/cell ratio and possibly sublethal conditions triggers a specific response leading to non-lytic membrane damage, nucleotidase release and Ado accumulation as an alternative mechanism of action of defensin, not necessarily related to its antimicrobial activity. Of course, there are several membrane parameters which are different among the strains and would influence peptide interactions [28], and a given peptide can have different mechanisms in different bacteria [29]; the results must therefore be considered cautiously, as indications that Ado accumulation is associated to 5’NT release induced by defensin, rather than to the lethality of the treatment.

In summary, the present report provides correlation evidence that 5’ nucleotidase activity is released from bacterial periplasmic space after defensin treatment and that this enzyme is responsible for AMP dephosphorylation, leading to the extracellular accumulation of Ado. Additional characterization of the supernatants from treated cultures, including a more complete enzymological study and immunoblotting analyses, can further validate our results and confirm the identity of the implied nucleotidase. Likewise, supplementary biochemical studies could help to elucidate a possible involvement of downregulated Ado transporters as a secondary event contributing to Ado accumulation.

From an ecological perspective, the manipulation of Ado signaling by bacteria in the mucosa can represent an advantageous mechanism of immune evasion and/or modulation of virulence factors, as has been suggested for other strains and species [30–33]. On the other hand, the implications for inflammatory processes in the gastrointestinal tract can be manifold, given the plurality of adenosine’s immunomodulatory effects, and the treatment of inflammatory GI disorders could benefit from the local production of Ado by specific members of the gut flora (i. e. the ones where nucleotidase can be found in the periplasm, such as *E*. *coli*), in the presence of optimal sub-lethal amounts of hBD-2. The main Ado receptor expressed in the intestinal epithelia, A_2B_ AR, has a EC_50_ of 24 μM [34, 35], at least one order of magnitude higher than the concentrations found in our assays. We speculate that the production of Ado *in situ* by bacteria in the intestinal mucosa might reach much higher local concentrations, due to the formation of microenvironments at the mucosal surface. In addition, we cannot exclude that in the presence of immune cells, especially during inflammatory processes, other AR could be activated by bacterial-derived Ado, e.g. A_1_, A_2A_ and A_3_, which have EC_50_ ranging from 0.3 to 0.7 μM [35]. *In vivo* evidence would be critical to confirm the physiological relevance of the phenomenon described in our study.

## Supporting Information

S1 File primary data set presented in figures Fig 1, Fig 2, and Fig 3. (PDF)

## References

[1] Ley RE, Lozupone C, Hamady M, Knight R, Gordon JI. Worlds within worlds: evolution of the vertebrate gut microbiota. Nat Rev Microbiol. 2008;6: 776–88. 10.1038/nrmicro1978 18794915 PMC2664199

[2] Backhed F, Ley RE, Sonnenburg JL, Peterson DA, Gordon JI, Bäckhed F. Host-bacterial mutualism in the human intestine. Science. 2005;307: 1915–1920. 10.1126/science.1104816 15790844

[3] Lee W-J, Hase K. Gut microbiota-generated metabolites in animal health and disease. Nat Chem Biol. 2014;10: 416–24. 10.1038/nchembio.1535 24838170

[4] Willyard C. Microbiome: Gut reaction. Nature. 2011;479: S5–S7. 10.1038/479S5a

[5] Huttenhower C, Kostic AD, Xavier RJ. Inflammatory bowel disease as a model for translating the microbiome. Immunity. 2014;40: 843–854. 10.1016/j.immuni.2014.05.013 24950204 PMC4135443

[6] Qin N, Yang F, Li A, Prifti E, Chen Y, Shao L, et al Alterations of the human gut microbiome in liver cirrhosis. Nature. 2014;513: 59–64. 10.1038/nature13568 25079328

[7] Kelly D, Conway S, Aminov R. Commensal gut bacteria: mechanisms of immune modulation. Trends Immunol. 2005;26: 326–33. 10.1016/j.it.2005.04.008 15922949

[8] Ganz T. Defensins: antimicrobial peptides of innate immunity. Nat Rev Immunol. 2003;3: 710–20. 12949495 10.1038/nri1180

[9] Ayabe T, Satchell DP, Wilson CL, Parks WC, Selsted ME, Ouellette AJ. Secretion of microbicidal alpha-defensins by intestinal Paneth cells in response to bacteria. Nat Immunol. 2000;1: 113–8. 11248802 10.1038/77783

[10] Bevins CL, Salzman NH. Paneth cells, antimicrobial peptides and maintenance of intestinal homeostasis. Nat Rev Microbiol. 2011;9: 356–68. 10.1038/nrmicro2546 21423246

[11] Hilchie AL, Wuerth K, Hancock REW. Immune modulation by multifaceted cationic host defense (antimicrobial) peptides. Nat Chem Biol. Nature Publishing Group; 2013;9: 761–8. 10.1038/nchembio.1393 24231617

[12] Fjell CD, Hiss JA, Hancock REW, Schneider G. Designing antimicrobial peptides: form follows function. Nat Rev Drug Discov. 2012;11: 37–51. 10.1038/nrd3591 22173434

[13] Easton DM, Nijnik A, Mayer ML, Hancock REW. Potential of immunomodulatory host defense peptides as novel anti-infectives. Trends Biotechnol. 2009;27: 582–90. 10.1016/j.tibtech.2009.07.004 19683819 PMC7114281

[14] Estrela AB, Rohde M, Gutierrez MG, Molinari G, Abraham WR. Human beta-defensin 2 induces extracellular accumulation of adenosine in *Escherichia coli* . Antimicrob Agents Chemother. 2013;57: 4387–4393. 10.1128/AAC.00820-13 AAC.00820-13 [pii] 23817371 PMC3754287

[15] Ochoa-Cortes F, Liñán-Rico A, Jacobson KA, Christofi FL. Potential for developing purinergic drugs for gastrointestinal diseases. Inflamm Bowel Dis. 2014; 20: 1259–87. 10.1097/MIB.0000000000000047 24859298 PMC4340257

[16] Chan ES, Cronstein BN. Molecular action of methotrexate in inflammatory diseases. Arthritis Res. 2002; 4: 266–273. 10.1186/ar419 12106498 PMC128935

[17] Estrela AB, Abraham WR. Adenosine in the inflamed gut: a Janus faced compound. Curr Med Chem. 2011;18: 2791–2815. doi:BSP/CMC/E-Pub/2011/ 203 [pii] 21649583 10.2174/092986711796011274

[18] Boudeau J, Glasser A, Masseret E, Joly B, Darfeuille-Michaud A. Invasive ability of an *Escherichia coli* strain isolated from the ileal mucosa of a patient with Crohn’s disease. Infect Immun.1999;67: 4499–4509. 10456892 10.1128/iai.67.9.4499-4509.1999 PMC96770

[19] Broad DF, Smith JT. *Escherichia coli* 5’-nucleotidase: purification, properties and its release by osmotic shock. J Gen Microbiol. 1981; 123: 241–247. 6275004 10.1099/00221287-123-2-241

[20] Okamura S, Maruyama HB, Yanagita T. Ribosome degradation and degradation products in starved *Escherichia coli*. VI. Prolonged culture during glucose starvation. J Biochem; 73: 915–922. 4578801 10.1093/oxfordjournals.jbchem.a130174

[21] Rinas U, Hellmuth K, Kang R, Segger A, Schlieker H. Entry of *Escherichia coli* into stationary phase is indicated by endogenous and exogenous accumulation of nucleobases. Appl Environ Microbiol. 1995;61: 4147–4151. 8534082 10.1128/aem.61.12.4147-4151.1995 PMC167726

[22] Watanabe K, Tomioka S, Tanimura K, Oku H, Isoi K. Uptake of AMP, ADP, and ATP in *Escherichia coli* W. Biosci Biotechnol Biochem. 2011;75: 7–12. 10.1271/bbb.100063 21228488

[23] Neu HC. The 5’-nucleotidase of *Escherichia coli*. I. Purification and properties. J Biol Chem. 1967;242: 3896–3904. 5341265

[24] Watanabe K, Fukumoto H, Isoi K. Intracellular localization of ATP:AMP phosphotransferase in *Escherichia coli* . Biochem Biophys Res Commun. 1986; 134: 527–531. 10.1016/s0006-291x(86)80452-1 3004452

[25] Neu HC. The 5’-nucleotidases (uridine diphosphate sugar hydrolases) of the Enterobacteriaceae. Biochemistry. American Chemical Society; 1968;7: 3766–3773. 10.1021/bi00850a059 4878706

[26] Glick J, Garber N. The intracellular localization of *Pseudomonas aeruginosa* lectins. J Gen Microbiol. 1983;129: 3085–3090. 10.1099/00221287-129-10-3085 6317795

[27] Bhatti AR, DeVoe IW, Ingram JM. The release and characterization of some periplasm-located enzymes of *Pseudomonas aeruginosa* . Can J Microbiol. NRC Research Press; 1976;22: 1425–1429. 10.1139/m76-211 184895

[28] Yeaman MR, Yount NY. Mechanisms of antimicrobial peptide action and resistance. Pharmacol Rev. 2003;55: 27–55. 12615953 10.1124/pr.55.1.2

[29] Powers J-PS, Hancock REW. The relationship between peptide structure and antibacterial activity. Peptides. 2003;24: 1681–91. 10.1016/j.peptides.2003.08.023 15019199

[30] Crane JK, Shulgina I. Feedback effects of host-derived adenosine on enteropathogenic *Escherichia coli* . FEMS Immunol Med Microbiol. 2009;57: 214–28. 10.1111/j.1574-695X.2009.00598.x 19751218 PMC2818178

[31] Greenwell-Wild T, Vázquez N, Sim D, Chatterjee D, Orenstein JM, Sharon M, et al *Mycobacterium avium* infection and modulation of human macrophage gene expression. J Immunol. 2002;169: 6286–6297. 10.4049/jimmunol.169.11.6286 12444135

[32] Thammavongsa V, Kern JW, Missiakas DM, Schneewind O. *Staphylococcus aureus* synthesizes adenosine to escape host immune responses. J Exp Med. 2009;206: 2417–2427. 10.1084/jem.20090097 19808256 PMC2768845

[33] Drygiannakis I, Ernst PB, Lowe D, Glomski IJ. Immunological alterations mediated by adenosine during host-microbial interactions. Immunol Res. 2011;50: 69–77. 10.1007/s12026-011-8207-0 21479929 PMC3361322

[34] Strohmeier GR, Reppert SM, Lencer WI, Madara JL. The A2b adenosine receptor mediates cAMP responses to adenosine receptor agonists in human intestinal epithelia. J Biol Chem 1995;270:2387–94. 7836474 10.1074/jbc.270.5.2387

[35] Fredholm BB, Irenius E, Kull B, Schulte G. Comparison of the potency of adenosine as an agonist at human adenosine receptors expressed in Chinese hamster ovary cells. Biochem Pharmacol 2001;61:443–8. 10.1016/S0006-2952(00)00570-0 11226378

