# Supplementary material for: Release of Periplasmic Nucleotidase Induced by Human Antimicrobial Peptide in E. coli Causes Accumulation of the Immunomodulator Adenosine
Source: PLoS One. 2015 Sep 15;10(9):e0138033. doi: 10.1371/journal.pone.0138033 (PMC4570785; doi:10.1371/journal.pone.0138033)
Supplement: S1 File — (PDF) [file pone.0138033.s001.pdf]

Fig 1A

Time	hBD-2 treated						Untreated					
	Extracellular [Ado] (µM)			Extracellular [AMP] (µM)			Extracellular [Ado] (µM)			Extracellular [AMP] (µM)		
	#1	#2	#3	#1	#2	#3	#1	#2	#3	#1	#2	#3
30 min	0.1893	0.2116	0.1963	0.1993	0.2220	0.2188	0.0445	0.0428	0.0389	0.0379	0.0388	0.0357
6h	0.5570	0.6213	0.5670	0.7485	0.7386	0.8628	0.2119	0.2325	0.2045	0.2833	0.2958	0.2715
24h	0.5813	0.7963	0.6648	0.4038	0.5274	0.4423	0.0183	0.0110	0.0138	0.9952	1.0494	1.0500
48h	1.1318	1.1205	1.2272	0.2569	0.2406	0.3050	0.1468	0.0173	0.0201	1.6064	1.5063	1.5951

Calibration Curves

[Ado] (µM)	MRM Resp.
0.029	14726
0.058	26523
0.117	47986
0.234	96019
0.468	190221
0.935	364304
1.87	685735
3.74	1273892

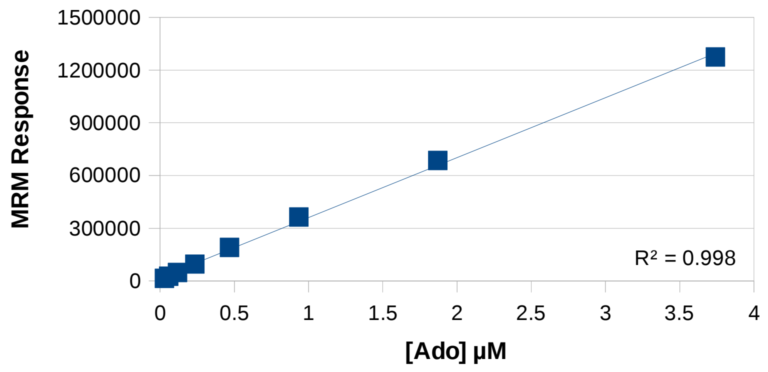

[AMP] (µM)	MRM Resp.
0.045	11015
0.09	21120
0.18	42676
0.36	86890
0.72	175075
1.44	351199
2.88	706448

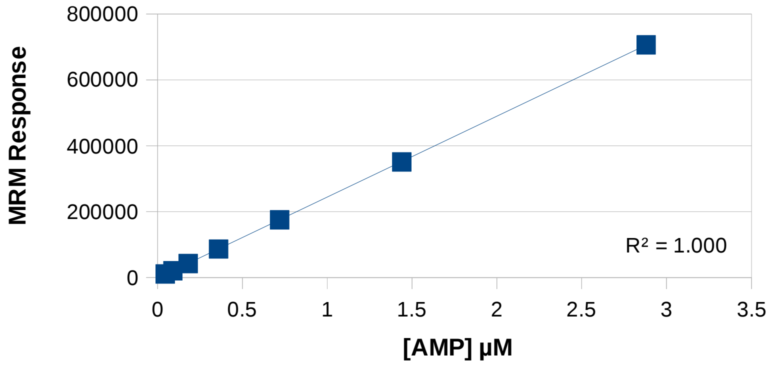

Fig 1B

	Extracellular [Ado] (μM)			
	hBD-2 treated		Untreated	
	#1	#2	#1	#2
**SN**	0.0188	0.0191	0.0039	0.0038
**SN+AMP**	0.1792	0.2247	0.0774	0.0944
**Cells**	0.0103	0.0091	0.0066	0.0069
**Cells+AMP**	0.0123	0.0113	0.0145	0.0143
**Medium**	0.0020	0.0020	ND	ND
**Medium+AMP**	0.0509	0.0355	ND	ND

Calibration Curve

[Ado] (μM)	MRM Resp.
0.029	19922
0.058	32895
0.117	67706
0.234	132971
0.468	261240
0.935	545639
1.87	1073174
3.74	2034062

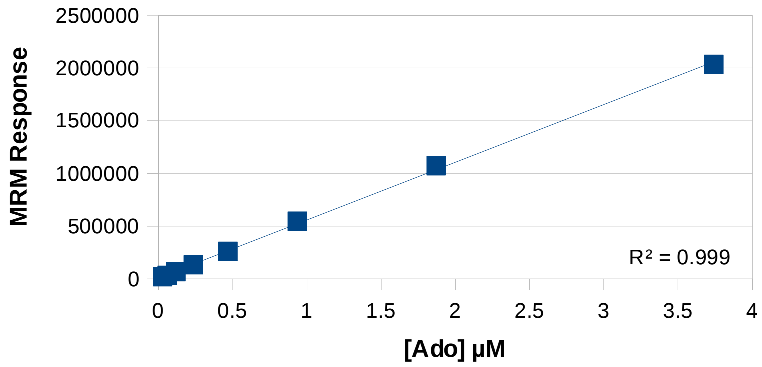

Fig 2A

	Absorbance 405 nm (pNPP dephosphorylation) Samples (SN)					Absorbance 405 nm (without pNPP)* Samples (SN)				
Time (h:m)	Before treatment	Untreated	AP	OS	Buffer*	Before treatment	Untreated	AP	OS	Buffer
0:30	0.104	0.108	0.115	0.13	0.103	0.079	0.088	0.086	0.085	0.079
4:00	0.103	0.122	0.162	0.284	0.102	0.08	0.086	0.088	0.087	0.079
7:30	0.103	0.144	0.226	0.404	0.102	0.08	0.086	0.088	0.088	0.079
11:00	0.105	0.17	0.296	0.488	0.102	0.08	0.086	0.089	0.088	0.079
14:30	0.104	0.2	0.368	0.541	0.102	0.08	0.086	0.089	0.089	0.079
18:00	0.104	0.229	0.429	0.57	0.101	0.08	0.086	0.09	0.088	0.079
21:30	0.105	0.26	0.478	0.586	0.102	0.081	0.086	0.089	0.089	0.079
25:00	0.104	0.288	0.513	0.591	0.101	0.08	0.087	0.09	0.089	0.08
28:29	0.105	0.315	0.534	0.59	0.101	0.08	0.087	0.09	0.09	0.08
31:59	0.104	0.338	0.547	0.586	0.101	0.081	0.087	0.09	0.089	0.08
35:29	0.105	0.356	0.552	0.581	0.102	0.081	0.088	0.09	0.09	0.08
38:59	0.105	0.37	0.552	0.574	0.102	0.081	0.087	0.09	0.091	0.08
42:30	0.105	0.382	0.548	0.567	0.102	0.081	0.088	0.09	0.09	0.079
46:00	0.105	0.387	0.543	0.559	0.101	0.081	0.088	0.091	0.09	0.08
48:00	0.106	0.39	0.54	0.554	0.102	0.081	0.088	0.091	0.091	0.08

* controls not displayed in the figure

Fig 2B

	Assay conditions		6h						18h					
	EDTA	Co2+	#1	#2	#3	#4	#5	#6	#1	#2	#3	#4	#5	#6
Extracellular [Ado] (µM)	-	-	0.1778	0.1838	0.1789	0.1849	0.1933	0.1989	1.3244	1.3439	1.3042	1.3109	1.3748	1.3796
	+	-	0.1993	0.2012	0.2008	0.1962	0.1963	0.1983	0.1435	0.0405	0.1996	0.1506	0.1208	0.1192
	-	+	0.2253	0.2266	0.2188	0.2239	0.2176	0.2279	3.2947	3.2890	3.2088	3.2222	1.0080	3.2891
	+	+	0.8010	0.8009	0.7935	0.7988	0.7941	0.7860	1.0058	0.9980	1.0277	1.0128	0.7635	0.7508
Extracellular [AMP] (µM)	EDTA	Co2+	#1	#2	#3	#4	#5	#6	#1	#2	#3	#4	#5	#6
	-	-	0.8737	0.8928	0.8671	0.8580	0.8917	0.9138	0.6497	0.6528	0.6303	0.6357	0.6589	0.6591
	+	-	0.4941	0.4959	0.4864	0.4951	0.5214	0.5155	0.4841	0.1488	0.6019	0.4904	0.5169	0.5051
	-	+	0.2445	0.2241	0.2247	0.2129	0.2499	0.2192	0.0328	0.0314	0.0324	0.0326	0.0312	0.0322
	+	+	0.0317	0.0312	0.0325	0.0312	0.0309	0.0303	0.0299	0.0301	0.0298	0.0304	0.0296	0.0300

Calibration Curves

[Ado] (µM)	MRM Resp.
0.029	12284
0.058	24551
0.117	48612
0.234	99528
0.468	205512
0.935	412816
1.87	840484
3.74	1683780

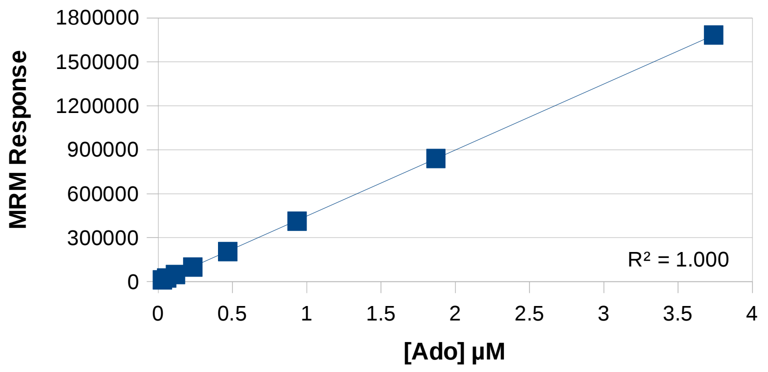

[Ado] (µM)	MRM Resp.
0.0225	6046
0.045	12192
0.09	24037
0.18	52153
0.36	117796
0.72	252103
1.44	520776
2.88	1068804

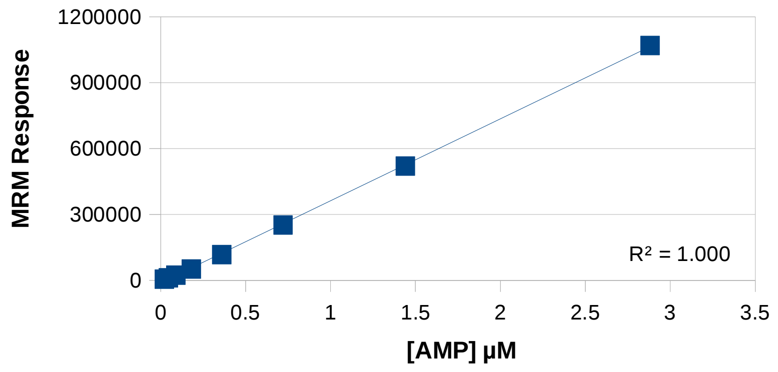

Fig 3A

	Extracellular [Ado] (µM)											
	hBD-2 treated						Untreated					
Strains	#1	#2	#3	#4	#5	#6	#1	#2	#3	#4	#5	#6
DSM 1116	0.1204	0.1164	0.1174	0.0940	0.1119	0.1142	0.0273	0.0280	0.0288	0.0288	0.0295	0.0282
Nissle 1917	0.0834	0.0830	0.0875	0.0839	0.0896	0.0877	0.0309	0.0315	0.0300	0.0304	0.0304	0.0299
7145A	0.0844	0.0862	0.0855	0.0850	0.0861	0.0869	0.0285	0.0286	0.0283	0.0279	0.0279	0.0280
HZI 2-6	0.1011	0.1026	0.1031	0.1005	0.1090	0.1133	0.0284	0.0290	0.0287	0.0294	0.0286	0.0289
LF82	0.0590	0.0610	0.0608	0.0482	0.0589	0.0580	0.0277	0.0274	0.0276	0.0275	0.0269	0.0274
DSM 19882	0.0329	0.0334	0.0296	0.0332	0.0322	0.0335	0.0413	0.0417	0.0390	0.0402	0.0396	0.0413

Calibration Curve

[Ado] (µM)	MRM Resp.
0.029	3594
0.058	7688
0.117	14962
0.234	30500
0.468	61595
0.935	125362
1.87	256562
3.74	535536

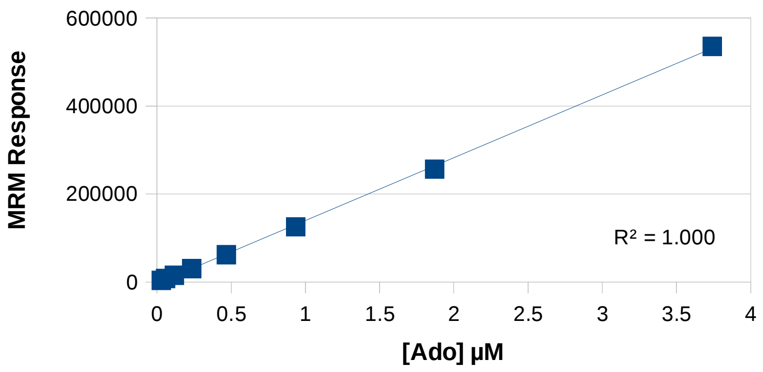

	Cell viability (CFU/mL)							
	Before treatment				hBD-2 (2h)			
Strains	#1	#2	#3	#4	#1	#2	#3	#4
DSM 1116	1.82E+08	1.93E+07	2.13E+07	1.84E+07	0.00E+00	0.00E+00	3.30E+04	5.00E+04
Nissle 1917	2.62E+07	2.74E+07	3.78E+07	1.51E+07	3.60E+05	1.30E+05	4.80E+05	3.20E+05
7145A	2.21E+08	1.31E+08	1.24E+08	1.43E+08	3.40E+07	2.80E+07	1.50E+08	2.41E+08
HZI 2-6	2.60E+07	2.40E+07	1.24E+07	3.27E+07	5.50E+05	4.20E+05	5.00E+05	4.60E+05
LF82	5.31E+07	2.06E+07	3.57E+07	1.89E+07	1.39E+06	1.13E+06	1.03E+06	1.26E+06
DSM 19882	1.04E+07	2.59E+07	4.75E+07	2.85E+07	3.70E+05	3.40E+05	4.50E+05	4.90E+05

Fig 3B

Time (h:m)	Absorbance 405 nm (pNPP dephosphorylation) Strains (SN)					Absorbance 405 nm (without pNPP)* Strains (SN)				
	DSM 1116	Nissle 1917	HZI 2-6	LF82	DSM 19882	DSM 1116	Nissle 1917	HZI 2-6	LF82	DSM 19882
0:00	0.116	0.118	0.141	0.141	0.111	0.103	0.097	0.098	0.105	0.122
0:30	0.119	0.117	0.125	0.117	0.109	0.096	0.093	0.095	0.091	0.094
2:30	0.157	0.152	0.174	0.159	0.111	0.097	0.094	0.095	0.092	0.094
4:30	0.201	0.189	0.226	0.206	0.113	0.096	0.093	0.095	0.092	0.094
6:30	0.247	0.232	0.277	0.254	0.114	0.096	0.093	0.095	0.093	0.094
8:30	0.294	0.273	0.324	0.3	0.115	0.096	0.093	0.095	0.093	0.093
10:30	0.341	0.315	0.369	0.345	0.117	0.096	0.093	0.094	0.093	0.094
12:30	0.386	0.356	0.409	0.385	0.118	0.097	0.094	0.095	0.093	0.094
14:30	0.427	0.396	0.445	0.422	0.12	0.096	0.094	0.095	0.093	0.094
16:30	0.466	0.433	0.476	0.455	0.122	0.097	0.093	0.094	0.094	0.095
18:30	0.498	0.469	0.501	0.48	0.123	0.097	0.093	0.094	0.093	0.094
20:30	0.525	0.499	0.523	0.504	0.125	0.096	0.093	0.095	0.093	0.094
22:30	0.548	0.527	0.54	0.525	0.127	0.096	0.094	0.094	0.093	0.094

* controls not displayed in the figure
